# Blueberry Muffin Syndrome and Hyperleukocytosis in a Newborn: A Diagnostic Challenge

**DOI:** 10.7759/cureus.52869

**Published:** 2024-01-24

**Authors:** Beatriz Teixeira, Ana Losa, Andreia Meireles, Ana Lachado, Isabel Couto Guerra, Susana Machado, Lídia Branco, Paulo Paulino, Catarina Lau, Natália Oliva-Teles, Carlos Mendes, Tereza Oliva, Liliana Pinho, Luísa Neiva, Elisa Proença

**Affiliations:** 1 Pediatrics Department, Centro Materno-Infantil do Norte, Centro Hospitalar Universitário de Santo António, Porto, PRT; 2 Neonatology Unit, Centro Hospitalar Tâmega e Sousa, Penafiel, PRT; 3 Pediatric Hematology Unit, Pediatrics Department, Centro Materno-Infantil do Norte, Centro Hospitalar Universitário de Santo António, Porto, PRT; 4 Dermatology Department, Centro Hospitalar Universitário de Santo António, Porto, PRT; 5 Immunology Department, Centro Hospitalar Universitário de Santo António, Porto, PRT; 6 Laboratorial Hematology Department, Centro Hospitalar Universitário de Santo António, Porto, PRT; 7 Clinical Hematology Department, Centro Hospitalar Universitário de Santo António, Porto, PRT; 8 Cytogenetics Unit, Centro de Genética Médica Doutor Jacinto Magalhães, Centro Hospitalar Universitário de Santo António, Porto, PRT; 9 Laboratorial Hematology Department, Instituto Português de Oncologia do Porto Francisco Gentil, Porto, PRT; 10 Pediatric Department, Instituto Português de Oncologia do Porto Francisco Gentil, Porto, PRT; 11 Neonatology Intensive Care Unit, Centro Materno-Infantil do Norte, Centro Hospitalar Universitário de Santo António, Porto, PRT

**Keywords:** cytogenetic analysis, acute myeloid leukemia, congenital leukemia, hyperleukocytosis, blueberry muffin syndrome

## Abstract

Blueberry muffin syndrome (BMS) in neonates, characterized by widespread nodular lesions, presents diagnostic challenges due to its diverse etiologies. Hyperleukocytosis, with leukocyte counts exceeding 100,000/μL, is a rare phenomenon associated with severe complications in neonates. Congenital leukemia (CL), a rare diagnosis within the first month of life, is linked to high mortality. This case report presents a unique case of BMS with hyperleukocytosis as the initial presentation of CL. A full-term male newborn, born after an uncomplicated pregnancy, except for Kell isoimmunization, with an Apgar score of 9/10, and an irrelevant family history, showed widespread purple nodules consistent with BMS at birth. Laboratory workup revealed mild anemia, hyperleukocytosis with immature granulocytes on peripheral blood (PB) smear, positive direct antiglobulin test, and elevated alanine aminotransferase and lactate dehydrogenase, without hyperbilirubinemia. Empirical antibiotics and hyperhydration were started, and the neonate was transferred to a level 3 neonatal intensive care unit for further evaluation. A comprehensive etiological investigation was conducted, comprising infectious, immunological, metabolic, and neoplastic factors. A skin nodule biopsy revealed an infiltrate of blast cells, indicative of leukemia cutis, and a bone marrow aspirate confirmed acute myeloid leukemia (AML). The patient successfully completed the NOPHO-DBH-2012 chemotherapy protocol at five months and remains in complete remission at nine months. This case report contributes to the literature by highlighting the diagnostic approach and management strategies for CL presenting with BMS and hyperleukocytosis. This case aims to enhance awareness and understanding of BMS as an initial manifestation of CL. Additionally, the challenges of treating leukemia in neonates, coupled with the lack of specific guidelines for this age group, further underscore the complexities in managing such patients.

## Introduction

Blueberry muffin syndrome (BMS) is an uncommon and non-specific clinical manifestation observed in neonates, distinguished by the presence of widespread nodular lesions displaying erythematous or violaceous hues. This syndrome can result from various etiologies, including congenital infections, severe fetal anemia, multifocal vascular anomalies, and neoplastic conditions [1]. Identifying the underlying causative factor of BMS can be challenging [2].

Leukocytosis is a well-recognized phenomenon in neonates, primarily attributed to physiological factors or infection-related responses. The occurrence of hyperleukocytosis, defined as leukocyte counts surpassing 100,000/μL, is atypical and warrants comprehensive investigation due to its potential to induce complications such as hyperviscosity and organ dysfunction. It is imperative to exclude potential underlying causes, including leukemia, transient abnormal myelopoiesis, and leukocyte adhesion defects [3].

Congenital leukemia (CL) is a rare diagnosis characterized by the onset of leukemia within the first month of life, constituting less than 1% of all childhood leukemia cases, and is associated with a high mortality rate. While the precise etiology and pathogenesis remain poorly understood, it is believed to arise from genetic mutations occurring during fetal development. Clinical manifestations frequently include hepatomegaly, splenomegaly, and skin lesions (leukemia cutis). Central nervous system (CNS) infiltration is observed in approximately 50% of cases. Newborns with hyperleukocytosis may also experience respiratory distress, cardiac issues, and renal failure [4].

The objective of this study is to present a case of BMS accompanied by hyperleukocytosis as the initial presentation of CL, with an emphasis on delineating the diagnostic approach and outlining management strategies.

## Case presentation

A full-term male newborn was delivered vaginally without complications in a level 2 hospital. The mother, aged 33 and healthy, had an uneventful pregnancy, except for the presence of Kell isoimmunization, to report one previous pregnancy without complications. Prenatal ultrasonography revealed no abnormalities, and maternal serological assessments indicated no signs of infection. The Group B Streptococcus test yielded a positive result, and peripartum prophylactic measures were effectively implemented. The Apgar score was 9/10 at the first and fifth minutes, and the birth weight was 3,045 g. The family history was irrelevant.

**Figure 1:**
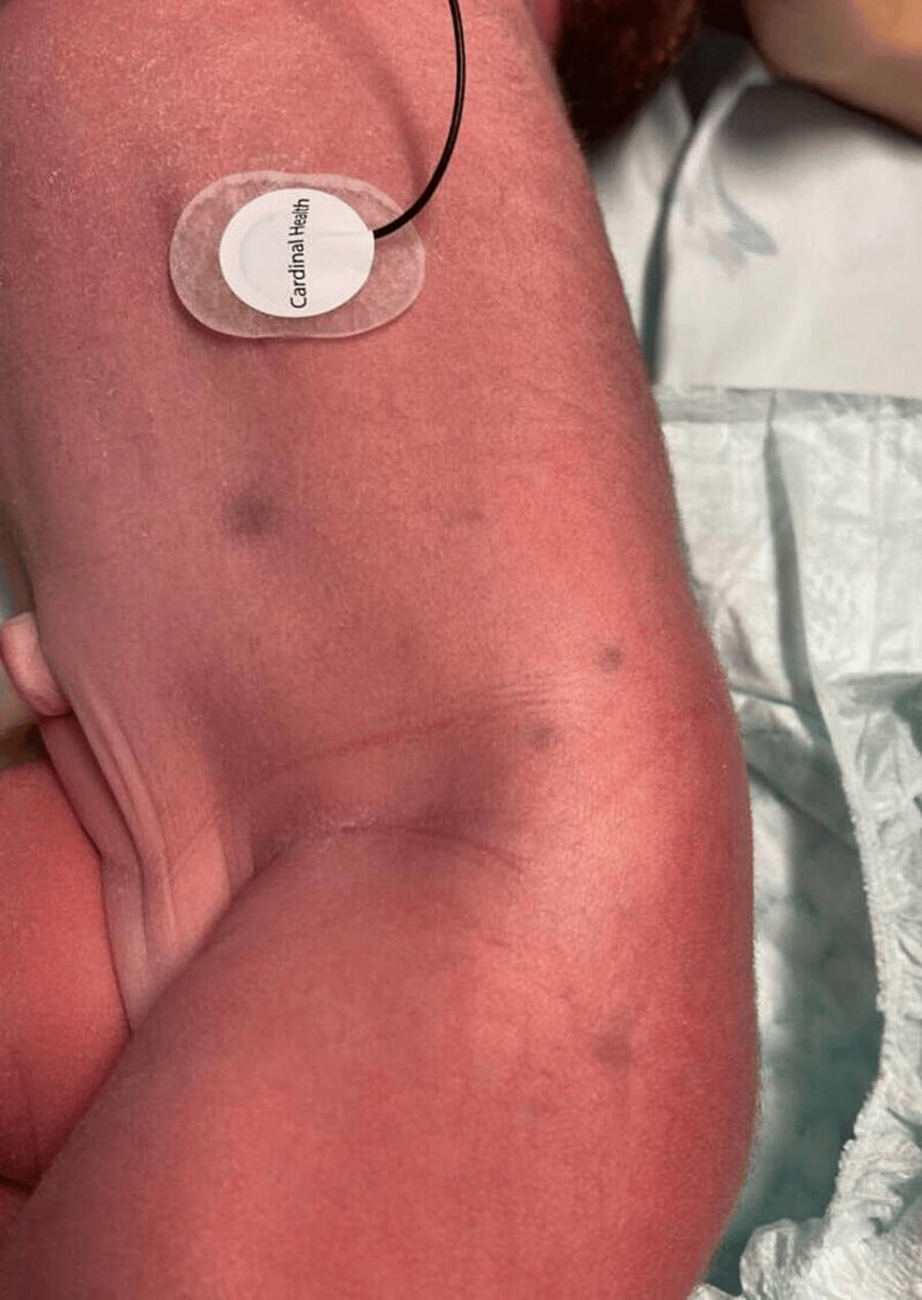
BMS lesions Physical examination after birth revealing widespread purple nodules, consistent with BMS.

At birth, physical examination revealed the presence of widespread purple nodules, each measuring up to 0.5 cm in diameter, which did not blanch upon the application of pressure. These nodules were observed on the face, trunk, abdomen, and limbs, as depicted in Figure 1, consistent with BMS. There were no other relevant findings.

**Figure 2:**
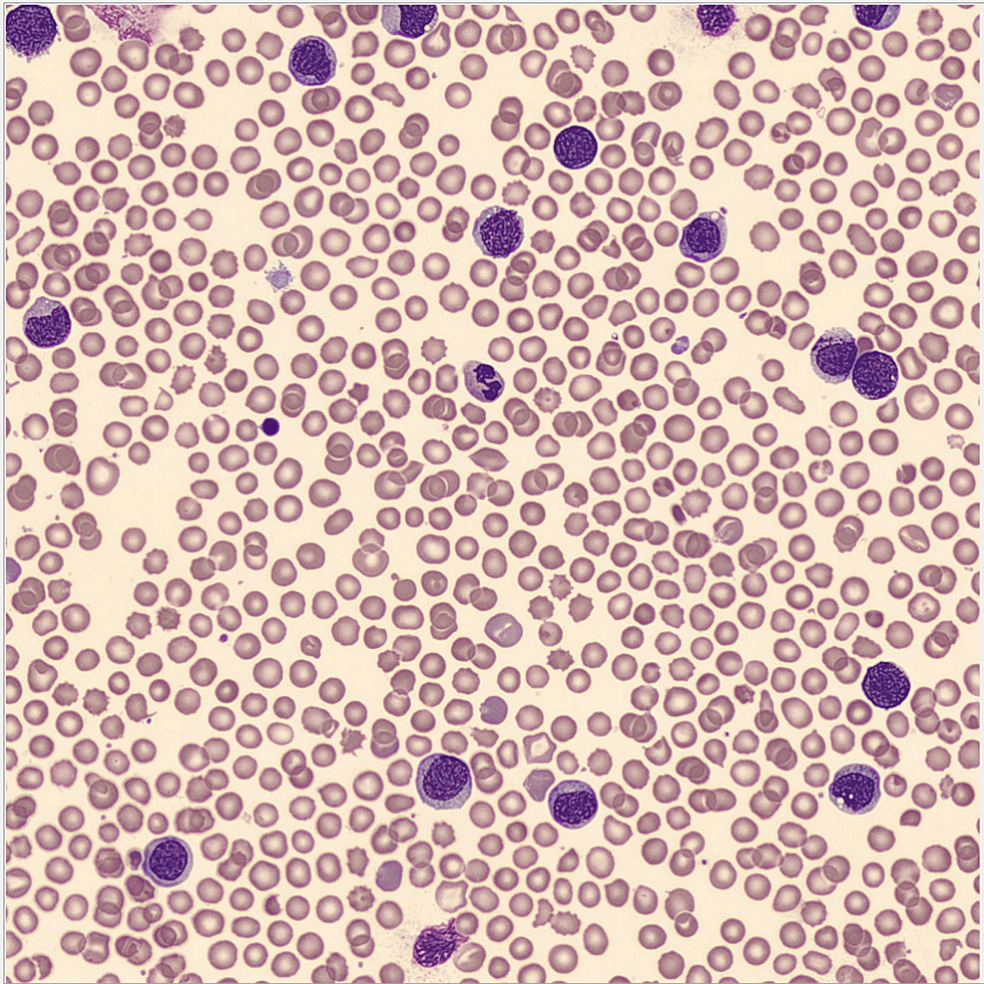
Peripheral blood smear Immature granulocytes in the peripheral blood smear, characterized by asynchrony in nucleus-to-cytoplasm maturation at the promyelocyte, myelocyte, and metamyelocyte stages.

Laboratory investigations (Table 1) unveiled the presence of mild anemia and hyperleukocytosis, along with a positive direct antiglobulin test. An analytical reassessment performed six hours after revealed an increase in leukocytosis, likewise the presence of immature granulocytes in the peripheral blood (PB) smear (Figure 2), characterized by asynchrony in nucleus-to-cytoplasm maturation at the promyelocyte, myelocyte, and metamyelocyte stages. Furthermore, elevated levels of alanine aminotransferase and lactate dehydrogenase were noted with normal bilirubin. C-reactive protein was negative.

**Table 1:** Laboratory results Laboratory results at birth, six hours of life, and later at day seven of life.

Test	Observed value at birth	Observed value at 6 hours of life	Observed value at day 7 of life	Reference range
Hemoglobin (g/dL)	12.6	13.2	10.2	13.5-21.5 g/dL
Hematocrit (%)	35.4	38.5	27.4	42.0-66,0%
Reticulocyte count (x10⁹/L)	--	--	44,600	50.0-100.0 x 10⁹/L
Platelet count (x10^3^/μL)	198.000	208.000	50.000	150-400 x 10^3^/μL
White blood cell count (x 10^3^/μL)	74.900	106.600	110.800	5.00-21.00 x 10^3^/μL
Peripheral blood smear	Large-sized cells with characteristics of immaturity - increased nucleus/cytoplasm ratio	Immature granulocytes (22% promyelocytes, 45% myelocytes, and 5% metamyelocytes)		
Lactate dehydrogenase (U/L)	--	1513	681	225-600 U/L
Alanine aminotransferase (U/L)	--	167	15	10-44 U/L
Total bilirubin (mg/dL)	3.3	5.1	--	0.20-1.00 mg/dL
C-reactive protein (mg/L)	<5	<5	10.41	0.0-5.0 mg/L
Direct antiglobulin test	Positive			

The neonate was promptly transferred to a level 3 neonatal intensive care unit (NICU) for further investigation and a comprehensive management approach.

Due to the presence of hyperleukocytosis (exceeding 100,000/μL), aggressive hydration therapy was initiated, with a maximum infusion rate of 200 mL/kg/day or 3,000 mL/m^2^/day. Empirical antibiotic treatment (ampicillin plus gentamicin) was also initiated.

Leukocyte counts exhibited fluctuations. Furthermore, there was a decline in hemoglobin levels (minimum of 9.5 g/dL on day six) and in platelet counts (minimum of 35,000/μL on day seven), without reticulocytosis. There were no other changes suggestive of hemolytic diseases in the newborn such as jaundice, hepatomegaly, or hyperbilirubinemia. Transfusion support was not required.

Radiographic assessments of the thoracoabdominal region and skeleton, cerebral ultrasound, echocardiogram, and ophthalmic examination did not reveal abnormalities. Abdominal ultrasound did indicate that the spleen size was at the upper limit of the normal range, with a normal liver span. A comprehensive etiological investigation was conducted, comprising infectious, immunological, metabolic, and neoplastic factors (Table 2).

**Table 2:** Etiological investigation Comprehensive etiological investigation conducted during hospitalization.

Test/ Exam	Relevant results
Thoracoabdominal and skeleton radiography	Without abnormalities
Abdominal ultrasound	Spleen size at the upper limit of the normal range, with normal liver span
Cerebral ultrasound	Without abnormalities
Echocardiogram	Without abnormalities
Ophthalmic examination	Without abnormalities
Blood cultures	Negative
TORCH infections screening	Negative
Quantitative analysis of adhesion molecules	Excluded LAD-1
Lysosomal acid lipase deficiency, Gaucher's disease, and congenital disorders of glycosylation screening	Excluded
Peripheral blood immunophenotyping	Presence of granulocytes with incomplete maturation
Skin lesion histology	Infiltrate of blast cells (leukemia cutis)
Bone marrow aspirate immunophenotyping	78.04% of monocytic blasts

Blood cultures, qualitative cytomegalovirus (CMV) urine polymerase chain reaction (PCR), rubella immunoglobulin M (IgM), toxoplasmosis IgM, herpes simplex 1 and 2, Epstein-Barr, and parvovirus B19 blood PCR were negative. Empirical antibiotic therapy was administered for a total of seven days, despite no evidence of bacterial infection.

Leukocyte adhesion deficiency type 1 was excluded, through quantitative analysis of adhesion molecules.

Metabolic causes, such as lysosomal acid lipase deficiency, Gaucher's disease, and congenital disorders of glycosylation, were excluded.

The most challenging part of the diagnostic process was ruling out neoplastic causes. Initial PB immunophenotyping yielded inconclusive results, indicating the presence of granulocytes with incomplete maturation, but failing to confirm the presence of neoplastic cells. A skin nodule biopsy and bone marrow (BM) aspirate were performed. Histological examination of the skin lesion revealed an infiltrate of blast cells, indicative of leukemia cutis. Concurrently BM immunophenotyping confirmed the presence of 78.04% monocytic blasts, consistent with a diagnosis of acute monoblastic/monocytic leukemia. This acute myeloid leukemia (AML) subtype was previously defined as AML-M5 according to the French-American-British (FAB) classification. Examination of cerebrospinal fluid (CSF) did not reveal the presence of pathological cells.

Cytogenetic analysis of PB revealed the existence of two distinct cell lines. The minority cell line exhibited a karyotype with 46 chromosomes, but with a chromosomal rearrangement, apparently balanced, involving the long arm of one of the X chromosomes and the long arm of one of chromosome 11. This rearrangement was understood as an insertional translocation, with breakpoints at Xq13, 11q23, and 11q13, accounting for 4.5% of cells, named mixed-lineage leukemia (MLL) gene or lysine [K]-specific methyltransferase 2A (KMT2A). The major cell line exhibited an apparently normal 46, XY karyotype. The final karyotype was proposed as 46,Y,?ins(X;11)(?q13;q23q13)[2]/46,XY(42). Fluorescence in situ hybridization (FISH) analysis of BM also confirmed the presence of a translocation involving the MLL gene, located at 11q23, in 97% of the analyzed cells, in accordance with the observations made in the PB analysis.

On day nine, in collaboration with the Pediatric Oncology team, treatment was initiated following the NOPHO-DBH-2012 chemotherapy protocol, including rasburicase.

Despite the risk stratification of tumor lysis syndrome (TLS) indicating an intermediate risk profile, no major complications were observed. However, due to the presence of hyperphosphatemia (maximum level of 2.66 mmol/L recorded on day 14), treatment with a phosphorus chelator was initiated on day 12, accompanied by vitamin D supplementation beginning on day 14.

Following the initiation of chemotherapy, the patient developed BM aplasia, prompting the initiation of prophylactic treatment with fluconazole and cefepime once the neutrophil count dropped below 500/μL. Additionally, palivizumab administrations were initiated.

On the 19th day of treatment, the patient manifested a respiratory syncytial virus infection, leading to episodes of apnea requiring non-invasive ventilation for five days. Subsequently, the patient encountered four episodes of febrile neutropenia, all of which were managed with empiric antibiotic regimens, resulting in favorable outcomes without the identification of any pathogen.

Oral feeding was initiated on day three and gradually increased. Given the mother's immunity to cytomegalovirus (CMV), regular scans were conducted to monitor for the presence of CMV in breast milk. Upon detecting a positive result, breast milk feeding was discontinued as a precautionary measure to prevent acquired infection.

The patient successfully completed the chemotherapy protocol at the age of five months, with minimal residual disease in the BM measuring less than 0.01%. Presently, at nine months of age, the patient remains in complete remission from leukemia. Both weight and height measurements remain below the 3rd percentile, despite experiencing reasonable weight gain. He also has normal psychomotor development.

## Discussion

CL is an exceedingly rare condition, constituting less than 1% of all childhood leukemia cases [4]. The estimated incidence of CL falls within the range of 4.3-8.6 cases per million live births, with only approximately 200 reported cases to date [5]. CL represents a diagnostic challenge due to its heterogeneous clinical presentation, requiring a high degree of clinical suspicion.

In the presented case, the diagnostic process was particularly challenging because typical symptoms, such as hepatosplenomegaly (present in 80% of cases), CNS involvement (present in 50%), and lymphadenopathy (present in 25%), were absent. Key diagnostic clues included hyperleukocytosis and the presence of BMS, with a skin biopsy proving essential to confirm the diagnosis by revealing leukemia cutis [1]. Leukemia cutis, occurring in up to 60% of CL cases, can often serve as the initial manifestation even without PB or BM involvement [4,5]. Nevertheless, when BMS is present, a complete blood count should be expeditiously performed [1].

The paucity of literature concerning the approach to CL, with most available data relying on individual case reports, underscores the considerable challenges inherent in managing this condition [1]. Furthermore, the vulnerability of neonatal patients and the necessity for age-adjusted treatment intensify the complexity of CL management.

Hyperleukocytosis always requires a quick approach. Hyperleucocytosis is the most consistent hematological feature of CL and requires early diagnosis and prompt intervention to prevent severe complications associated with blast infiltration and leukostasis, including hemorrhagic and thromboembolic events, acute respiratory failure, pulmonary hemorrhage, CNS or splenic infarction, and renal failure [3]. Supportive management should include hyperhydration to reduce blood viscosity [3]. In our patient, hyperleukocytosis was promptly identified and managed, resulting in the absence of severe complications.

Additionally, neonates with CL often present with anemia and/or thrombocytopenia at birth, which may worsen in the first hours or days of life, requiring close monitoring of blood counts [4]. In the presented case, considering the anti-Kell isoimmunization, it was also imperative to rule out hemolytic disease in the newborn.

During chemotherapy, the risk of TLS remains a concern, especially due to immature renal function [4]. In addition to hydration, the use of rasburicase, an enzyme that catalyzes the breakdown of uric acid into more soluble and less toxic products, appears to reduce this risk [4]. Prophylactic antibiotics and antifungal drugs are essential to prevent severe infections in this vulnerable population. Given the scarcity of specific protocols for newborns, selecting prophylactic measures was a challenge.

In CL, AML is more common than acute lymphoblastic leukemia (ALL), representing 56-64% of cases [4,5]. Among AML subtypes, the FAB classification M5 is the most prevalent (comprising 60% of patients). The rearrangement of the MLL gene, known as the KMT2A gene, at chromosome band 11q23, is the most frequent chromosomal aberration, affecting up to 93% of infants [6]. Despite the association of MLL gene rearrangements with poor prognosis [7] and a negative impact on complete remission, overall survival, and event-free survival [6,8], some patients, such as the one presented here, may respond well to treatment and achieve complete remission [8]. CL genetic changes are usually sporadic mutations that occur during fetal development.

CL is associated with a high mortality rate, with up to 74% of patients succumbing to the disease within two years of diagnosis [5,9]. Furthermore, CL is associated with an increased risk of treatment-related mortality and a high rate of relapse when compared to older children. Nevertheless, some studies suggest that chemotherapy for CL can yield good outcomes [4], such as our patient, who remains in complete remission.

Therefore, the authors recommend that, in the presence of BMS associated with hyperleukocytosis, it becomes imperative to simultaneously achieve two objectives: promptly managing hyperleukocytosis to prevent its complications in this age group and persisting in a comprehensive exploration of potential causes, never neglecting neoplastic etiology, even when the initial study is inconclusive in identifying neoplastic cells.

## Conclusions

In conclusion, this case underscores the diagnostic challenges and complexity associated with CL. Heightened awareness among healthcare providers regarding the manifestations of CL is crucial, as early diagnosis and intervention can have a profound impact on patient outcomes. BMS, although caused by various etiologies, can be a manifestation of CL. Considering that it may be the only finding upon objective examination, its identification and subsequent investigation are of paramount importance.

While there is limited available data on the optimal treatment strategies for CL, this case emphasizes the importance of a multidisciplinary approach to effectively diagnose and manage this complex condition. Given the scarcity of literature on congenital AML, sharing individual case reports within the medical community can serve as valuable learning opportunities, improving the collective knowledge of this rare condition.

## References

[1] Koster SB, Vinke ME, van den Bos C, van Heel WJ, Kranendonk ME, Natté R, van Tuyll van Serooskerken AM (2023). A case report of a blueberry muffin baby caused by congenital self-healing indeterminate cell histiocytosis. BMC Pediatr.

[2] Clark EE, Walton M, Chow LM, Boyd JT, Yohannan MD, Arya S (2023). Disseminated juvenile xanthogranuloma with a novel MYH9-FLT3 fusion presenting as a blueberry muffin rash in a neonate. AJP Rep.

[3] Parvez Y, Mathew AG (2014). Hyperleukocytosis in newborn: a diagnosis of concern. Indian J Hematol Blood Transfus.

[4] Roberts I, Fordham NJ, Rao A, Bain BJ (2018). Neonatal leukaemia. Br J Haematol.

[5] Green K, Tandon S, Ahmed M (2021). Congenital acute myeloid leukemia: challenges and lessons. A 15-year experience from the UK. Leuk Lymphoma.

[6] Huang S, Yang H, Li Y (2016). Prognostic significance of mixed-lineage leukemia (MLL) gene detected by real-time fluorescence quantitative pcr assay in acute myeloid leukemia. Med Sci Monit.

[7] El Chaer F, Keng M, Ballen KK (2020). MLL-rearranged acute lymphoblastic leukemia. Curr Hematol Malig Rep.

[8] Yang W, Qin M, Jia C (2022). Pediatric acute myeloid leukemia patients with KMT2A rearrangements: a single-center retrospective study. Hematology.

[9] Kaleta K, Kłosowicz A, Juśko N, Kapiñska-Mrowiecka M (2022). Blueberry muffin baby syndrome. A critical primary sign of systemic disease. Postepy Dermatol Alergol.

